# Malaysian endophytic fungal extracts-induced anti-inflammation in Lipopolysaccharide-activated BV-2 microglia is associated with attenuation of NO production and, IL-6 and TNF-α expression

**DOI:** 10.1186/s12906-015-0685-5

**Published:** 2015-06-06

**Authors:** Azzeme Harun, Sharmili Vidyadaran, Siong Meng Lim, Anthony L J Cole, Kalavathy Ramasamy

**Affiliations:** Faculty of Pharmacy, Universiti Teknologi MARA (UiTM), 42300 Bandar Puncak Alam, Selangor Darul Ehsan Malaysia; Collaborative Drug Discovery Research (CDDR) Group, Pharmaceutical and Life Sciences Community of Research, Universiti Teknologi MARA (UiTM), 40450 Shah Alam, Selangor Darul Ehsan Malaysia; Neuroinflammation Group, Immunology Laboratory, Faculty of Medicine and Health Sciences, Universiti Putra Malaysia, 43400 UPM Serdang, Selangor Darul Ehsan Malaysia; School of Biological Sciences, University of Canterbury, Private Bag 4800, Christchurch, New Zealand

**Keywords:** Endophyte, Fungi, Inflammation, Nitric oxide, CD40, Cytokines

## Abstract

**Background:**

Excessive production of inflammatory mediators such as nitric oxide (NO) and proinflammatory cytokines like tumour necrosis factor-alpha (TNF-α) from activated microglia contributes to uncontrolled inflammation in neurodegenerative diseases. This study investigated the protective role of five endophytic extracts (HAB16R12, HAB16R13, HAB16R14, HAB16R18 and HAB8R24) against LPS-induced inflammatory events *in vitro*. These endophytic extracts were previously found to exhibit potent neuroprotective effect against LPS-challenged microglial cells.

**Methods:**

The effects of these fungal endophytic extracts against nitric oxide (NO), CD40 phenotype and, pro- and anti-inflammatory cytokine production in lipopolysaccharide (LPS)-stimulated BV2 microglia cells were examined using commercially available assay kits, immunophenotyping and flow cytometry, respectively.

**Results:**

Microglia pre-treated with the five endophytic extracts (0.1 mg/mL) reduced NO production without compromising cell viability. Whilst CD40 expression in LPS-stimulated microglia was not significantly different with or without the influence of endophytic extracts, expression of the proinflammatory cytokines, IL-6 and TNF-α in LPS-stimulated microglia was significantly (*P* < 0.05) inhibited by these endophytic extracts.

**Conclusions:**

The outcomes suggest that the neuroprotective effect of the fungal endophytic extracts is likely mediated through supression of neuroinflammation. To our knowledge, this is the first report of the effect of a fungal endophytic extract in controlling inflammation in BV2 microglia cells.

**Electronic supplementary material:**

The online version of this article (doi:10.1186/s12906-015-0685-5) contains supplementary material, which is available to authorized users.

## Background

Inflammation in the brain was previously observed only as a reactive response to neuronal damage [1]. There is increasing evidence, however, indicating that inflammation also causes neuronal death and damage [2] which in turn leads to neurodegenerative diseases such as Alzheimer’s disease, Parkinson’s disease and multiple sclerosis. Amongst the various cell types associated with inflammation-mediated neurodegeneration, microglia have been implicated as vital components of the immunological insult to neurons [3].

Microglia account for approximately 12 % of cell population [4] in the central nervous system (CNS). They are brain-specific macrophages that provide trophic support and maintains homeostasis in healthy tissue [5]. During brain infection or injury, microglia become activated and up-regulate a variety of surface receptors which include the major histocompatibility complex and complement receptors [1]. They also release various pro-survival neurotrophic factors like brain-derived neurotrophic factor (BDNF), neurotrophin-3 (NT-3) and nerve growth factor (NGF) [6], and proinflammatory molecules such as tumour necrosis factor alpha (TNF-α), interleukin 1 beta (IL-1β) and free radicals nitric oxide (NO) and superoxide anion. All these may lead to a beneficial inflammatory response and recovery. Nevertheless, over-activation of microglia can cause neuronal toxicity and death, exacerbating neurodegenerative diseases [7]. It is now generally accepted that microglial activation is involved in the initiation and progression of multiple neurodegenerative diseases [8]. Therefore, early detection of microglial activation and anti-inflammatory therapy may delay or halt disease progression before irreversible damage and clinical symptoms occur.

Medicinal plants, plant extracts and isolated secondary metabolites have traditionally been used to treat a variety of diseases including those associated with acute and chronic inflammation [9]. Endophytic fungi which form symbiotic associations with plants and often produce protective compounds for their hosts, however, remain one of the most unexplored group of microorganisms for their protective effects against inflammation [10]. In a recent report,, five extracts (HAB16R12, HAB16R13, HAB16R14, HAB16R18 and HAB8R24) with potent BACE 1 inhibitory activity were identified from amongst 212 endophytic extracts [11]. These extracts showed potential to be developed into therapeutics that can be used for treatment against neurodegenerative diseases. Given the relationship between BACE1 expression and inflammation [12], the present study investigated for the possible role of these extracts with BACE1 inhibitory effect in modulating *in vitro* inflammatory responses of LPS-activated BV2 microglia cells.

## Methods

### Fungi

Fungal endophytes were obtained from the culture collection of the Collaborative Drug Discovery Research (CDDR) Group, Faculty of Pharmacy, Universiti Teknologi MARA (UiTM), Malaysia. They were previously isolated from medicinal plants at Kuala Pilah rainforest in Negeri Sembilan, Malaysia [13]. Axenic cultures were maintained on potato dextrose agar (PDA, Oxoid, Basingstoke, Hampshire, England) plates and incubated at 28 °C for 14 days. Five endophytic fungi (HAB16R12, HAB16R13, HAB16R14, HAB16R18 and HAB8R24) cultures were extracted and assessed for their bioactivity. These fungi were all isolated from the roots of *Cinnamomum porrectum* [11, 13]. The ITS of HAB10R12, HAB16R13, HAB16R14, HAB16R18 and HAB8R24 were found to be 586–593 bp in length. A BLAST search of the ITS of all five isolates revealed that they were 98–99 % identical to *Cytospora rhizophorae* [11]. Their GenBank accession numbers were JN083836, HQ336045, JN083837, JN083838 and JN083839, respectively.

### Semipolar extraction of fungal cultures

Semipolar extraction was performed as described by NA Hazalin, K Ramasamy, SM Lim, IA Wahab, AL Cole and AB Abdul Majeed [14]. Briefly, after 14 days of incubation, 10 plates of each fungus were transferred into a conical flask (500 mL) and homogenised (Kika Labortechnik T25, Staufen, Germany). Ethyl acetate (200 mL) was added and left to stir overnight at room temperature. The mixture was filtered through Whatman No.1 filter paper, after which sodium sulphate (Merck, Darmstadt, Germany) was added to remove the aqueous layer within the mixture. The mixture was then transferred to a round bottom flask (500 mL) and evaporated to dryness. The resultant extract was dissolved in 1 mL dimethyl-sulfoxide (DMSO) (Sigma, St Louis, Missouri, USA) and stored at -20 °C until further use.

### BV2 microglia cell line

The BV2 microglia cell line was provided by Dr. Sharmili Vidyadaran (Universiti Putra Malaysia, UPM) and maintained in Dulbecco’s Modified Eagle Medium (DMEM) (Gibco, Grand Island, New York, USA) with 5 % heat-inactivated foetal bovine serum, 100 U/mL penicillin, 100 μg/mL streptomycin, 1 mL/L gentamycin (all Invitrogen, Grand Island, New York, USA), 6.25 μg/mL insulin (Sigma, St Louis, Missouri, USA), 1 × non-essential amino acids (Sigma, St Louis, Missouri, USA) and 1.5 g/L sodium bicarbonate (NaHCO_3_) (Sigma, St Louis, Missouri, USA) at 37 °C with 5 % CO_2_ unless otherwise stated [15].

### Griess assay for Nitric Oxide (NO) production

The Griess assay measures the level of accumulated nitrite (NO_2_^−^), a metabolite of nitric oxide (NO) in culture supernatant using the Griess reagent [2.5 % phosphoric acid (Merck, Darmstadt, Germany), 1 % sulphanilamide (Sigma, St Louis, Missouri, USA) and 0.1 % napthy ethylene diamine dihydrochloride (Sigma, St Louis, Missouri, USA)]. Cells were plated in 96-well microtiter plates at a density of 1 × 10^5^ cells per well and incubated for 24 h. Cells were then treated with medium supplemented with endophytic extracts (0.1–1000 μg/mL) and after 24 h, the media was changed to 250 μL DMEM (without phenol red) per well and the standard iNOS inhibitor, N-nitro-L-arginine methyl ester (L-NAME) (250 μM) (Sigma, St Louis, Missouri, USA) added to designated wells. The cells were then stimulated with 1 μg/mL lipopolysaccharide (LPS). Supernatants were collected at 18, 24 and 48 h for nitrite (NO_2_^−^) production assay. An equal volume of Griess reagent was mixed with cell culture supernatants and color development assessed at absorbance 530 nm with a microplate reader (MRX® II, Dynex Technologies, Chantilly, Virginia, USA). The amount of NO_2_^−^ in the samples was calculated from a standard curve (0–100 μM) of freshly prepared sodium nitrite. Cell viability was determined using the MTT reduction assay [16] after 24 h and 48 h.

### Cell viability assay

Cells were plated in 96-well microtiter plates at a density of 1 × 10^5^ cells per well and incubated for 24 h. Cells were then treated with medium supplemented with endophytic extracts (0.1–1000 μg/mL) for 24 h. The media was changed to 250 μL DMEM per well and the cells were stimulated with 1 μg/mL lipopolysaccharide (LPS) for 24 h and 48 h, respectively, after which MTT assay was performed. Under dark conditions, 10 μL of 3-(4,5-dimethylthiazol-2-yl)-2,5-diphenyltetrazolium bromide (MTT) (Sigma, Schnelldorf, Germany) solution (5 mg/mL in PBS) was added to BV2 cells in each 96 well plate containing 100 μL media and incubated for 3 h. MTT solution was removed and 200 μL dimethyl sulfoxide (DMSO) was added to dissolve the formazan dye. The plate was shaken for 3 min and absorbance determined at 570 nm using a microplate reader (MRX® II, Dynex Technologies, Chantilly, Virginia, USA).

### Immunophenotyping

BV2 microglia cells were seeded in a 12-well plate at a seeding density of 2 × 10^5^ cells per well, incubated for 24 h and then treated with endophytic extracts at 0.1 mg/mL (extracts exhibited not more than 30 % cell kill at this concentration) and incubated for 24 h. Cells were then stimulated with LPS (1 μg/mL) and incubated for 18, 24 and 48 h under the same conditions. Cells were harvested by trypsinisation with 0.25 % trypsin (200 μL per well) for 5 min. Media (600 μL) was added to stop the trypsinisation and then transferred into the fluorescence activated cell sorting (FACS) tube, spun down at 2000 rpm for 5 min and cell pellet collected. The cell pellet was washed in 1 mL PBS/0.2 % bovine serum albumin (BSA) and spun down at 2000 rpm for 5 min. Under dark conditions, anti-CD40-FITC (BD Biosciences) was added to the pellet and subsequently incubated at 4 °C for 30 min. Isotype-matched antibodies to FITC was used as control to determine non-specific staining. BSA (0.2 %in PBS; 1 mL) was added and mixture spun down at 2000 rpm for 5 min. The resultant cell pellet was resuspended in 500 μL 0.2 % BSA in PBS and analysed using a FACSCalibur cytometer (BD Biosciences, San Jose, CA). A total of 10,000 cells (events acquired) were selected for analysis.

### Cytokine assay

BV2 microglia cells were seeded onto a 6-well plate at a density of 2 × 10^5^ cells per well and incubated for 24 h. The cells were then treated with 0.1 mg/mL endophytic extracts and incubated for 24 h. Treated cells were stimulated with LPS (1 μg/mL) and incubated for 24 and 48 h. The supernatant was collected and assayed for interleukin (IL)-6, IL-10, IL-12p70, tumor necrosis factor alpha (TNF-α), monocyte chemoattractant protein-1 (MCP-1) and interferon-gamma (IFN-γ). The Becton Dickson Cytometric Bead Array (CBA) Mouse Inflammation Kit (BD Biosciences, San Diego, California, USA) which captures a soluble analyte or set of analytes with beads of known size and fluorescence was employed and analysed using BD FACSCalibur flow cytometer (BD Biosciences, San Diego, California, USA) and BD CellQuest Pro software (BD Biosciences, San Diego, California, USA). Briefly, 50 μL of each test sample supernatant was transferred into assay tubes. The mixed capture beads (CB, 50 μL) was added to the assay tubes containing test samples and to the control tubes [50 μL of the mouse inflammation standards (MIS) dilutions]. Then, 50 μL of the mouse inflammation PE detection reagents were added to the entire test assay tubes and incubated for 2 h at room temperature in darkness. Wash buffer (1 mL) was added to each assay tube and centrifuged at 200 × g for 5 min. The bead pellet was resuspended in 300 μL wash buffer and analysed using FACSCalibur cytometer (BD Biosciences, San Jose, CA). The BD FACSCalibur flow cytometer is a four-color, dual laser and bench top flow cytometry system that provides both cell analysis and sorting. For intra-assay precision, ten replicates of each of the three different levels of IL-6, IL10, MCP-1, IFN-γ, TNF-α and IL-12p70 were tested and for inter-assay precision, three different levels of IL-6, IL10, MCP-1, IFN-γ, TNF-α and IL-12p70 (80, 625, 2500 pg/mL) were tested in four experiments. The detection limit of IL-6, IL10, MCP-1, IFN-γ, TNF-α and IL-12p70 is 5, 17.5, 52.7, 2.5, 7.3 and 10.7 pg/mL, respectively.

### Statistical analysis

Differences in the anti-inflammatory effects of the extracts after treatment were evaluated using the One-Way ANOVA procedure of the SPSS version 17.0. When there was a difference, the LSD’s post hoc test was used to identify pairs that differed significantly. Differences were considered as significant at *P* < 0.05.

## Results

### Pre-treatment of BV2 microglial cells with endophytic extracts offers protection against oxidative stress through attenuation of LPS-induced NO production

**Fig. 1:**
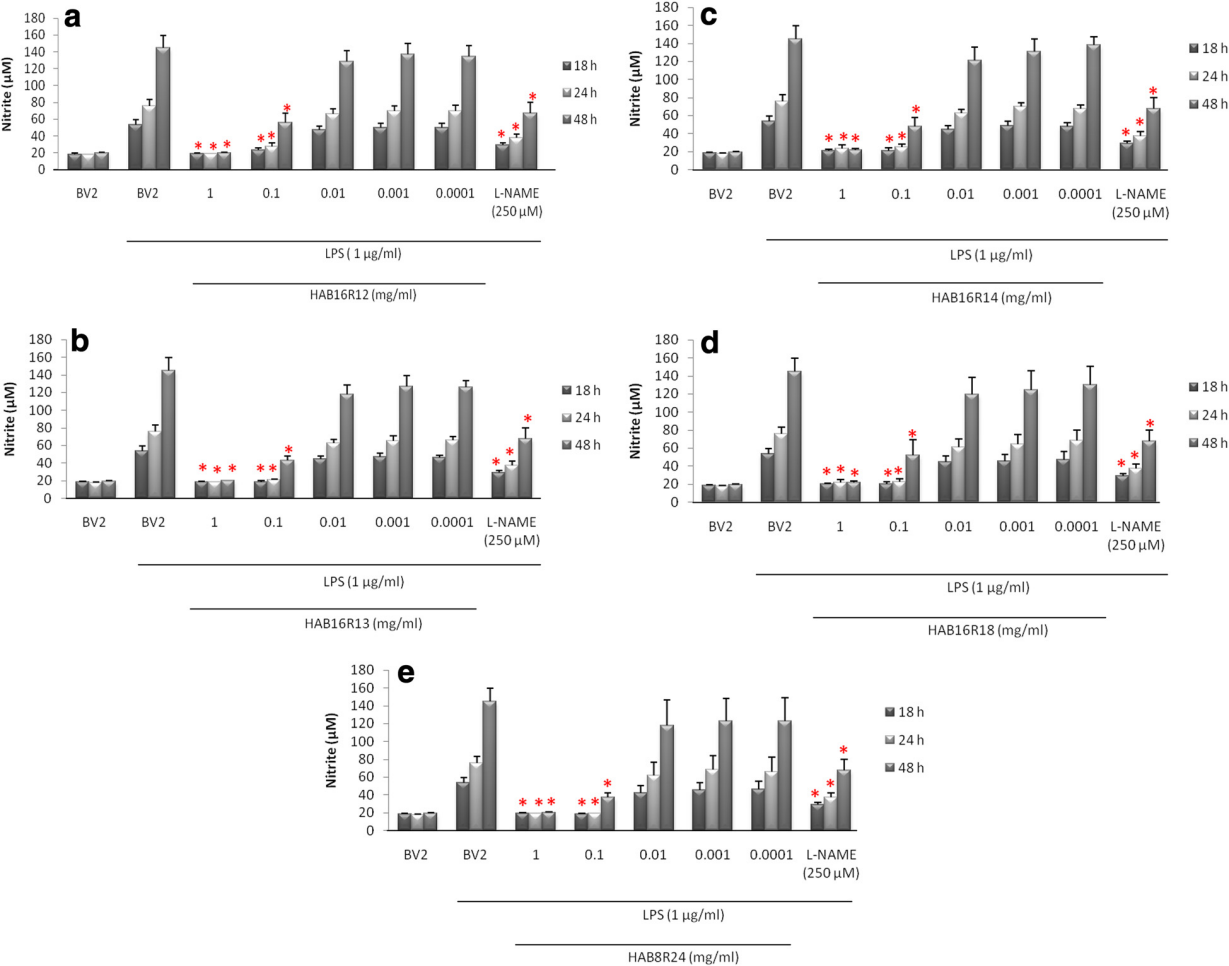
Effects of pre-treatment with extracts HAB16R12 (**a**), HAB16R13 (**b**), HAB16R14 (**c**), HAB16R18 (**d**) and HAB8R24 (**e**) on NO production in LPS-stimulated BV2 microglia cells. Data are expressed as means ± SEM of three independent experiments. *P < 0.05, significantly different when compared to LPS-treated control (BV2)

NO (~20 μM) was present in unstimulated BV2 microglia cells (resting cells) at all time points (Fig. 1). The basal level of NO was markedly increased to 54 μM (2.7 fold), 76 μM (3.8 fold) and 145 μM (7.3 fold) after exposure to LPS for 18 h, 24 h and 48 h, respectively (Fig. 1). Pre-treatment of BV2 with all five extracts at 0.1 mg/mL and 1.0 mg/mL significantly reduced subsequent LPS-induced NO production (Fig. 1; *P* < 0.05). For pre-treatment at 0.1 mg/mL, NO was significantly reduced by approximately 57.5–66.1 %, 64.2–74.7 % and 61.6–74.4 % following exposure to LPS for 18, 24 and 48 h, respectively (*P* < 0.05). Pre-treatment with extracts at lower concentrations (0.01–0.0001 mg/mL), however, reduced NO production by only 6.6–21.8 %, 8.0–19.7 % and 4.8–18.8 % following exposure to LPS for 18 h, 24 h and 48 h, respectively. For pre-treatment at 1.0 mg/mL NO production was significantly (*P* < 0.05) reduced by approximately 61.5–65.9 %, 68.9–76.0 % and 85.0–86.4 % following exposure to LPS for 18 h, 24 h and 48 h, respectively. The iNOS inhibitor, L-NAME (250 μM) that was added to BV2 simultaneously with LPS, significantly (*P* < 0.05) reduced NO production by 46.0 %, 50.7 % and 53.4 % at 18 h, 24 h and 48 h, respectively. The present study found the NO lowering effect of pre-treatment with endophytic extracts (0.1–1 mg/mL) to be either comparable to (at 0.1 mg/mL) or otherwise better (at 1.0 mg/mL) than that of standard nitric oxide synthase (NOS) inhibitor, L-NAME. It is noteworthy that the protective effects of pre-treatment with endophytic extracts as observed had no effect on basal NO release by microglia (Additional file 1: Figure S1).

### Pre-treatment with endophytic extracts were not cytotoxic against BV2 microglia cells at concentrations < 1.0 mg/ml

**Fig. 2:**
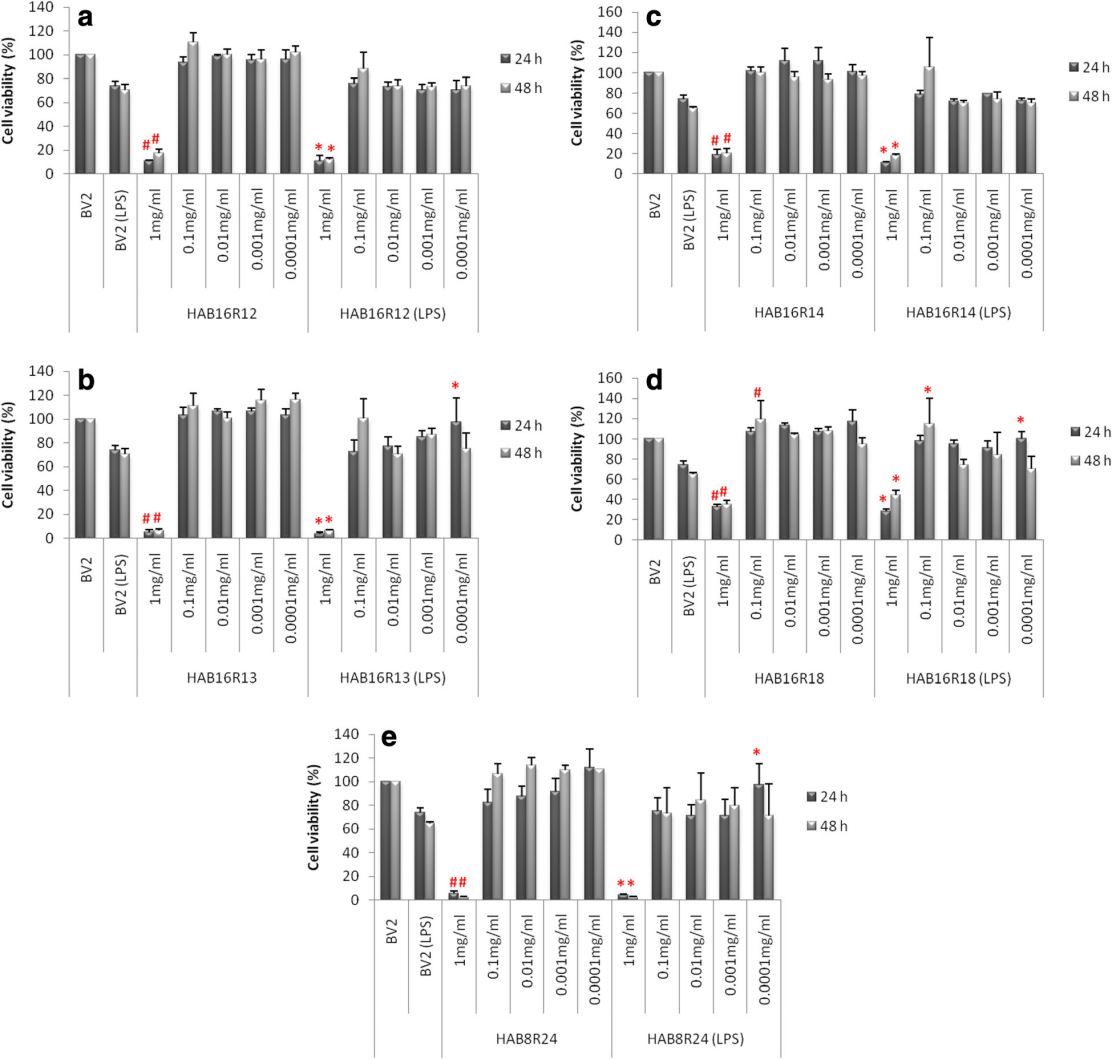
Effects of pre-treatment with extracts HAB16R12 (**a**), HAB16R13 (**b**), HAB16R14 (**c**), HAB16R18 (**d**) and HAB8R24 (**e**) on BV2 microglia cell viability. Each value is mean percentage ± SEM of three independent experiments. #*P <* 0.05, when compared to control group (BV2 resting), *P < 0.05, when compared to the LPS-treated group

Except for 1.0 mg/mL (<40 % cell viability; *P* < 0.05), pre-treatment with all five extracts did not significantly affect the cell viability of BV2 microglia cells between concentrations of 0.0001–0.1 mg/mL, with or without LPS (Fig. 2). As such, the highest subtoxic concentration of the extract (0.1 mg/mL), which exhibited good NO lowering effect without affecting cell viability, were chosen for subsequent anti-inflammatory study.

### Endophytic extracts altered CD40 expression on unstimulated BV2 microglia cells but not their stimulated counterparts

**Fig. 3:**
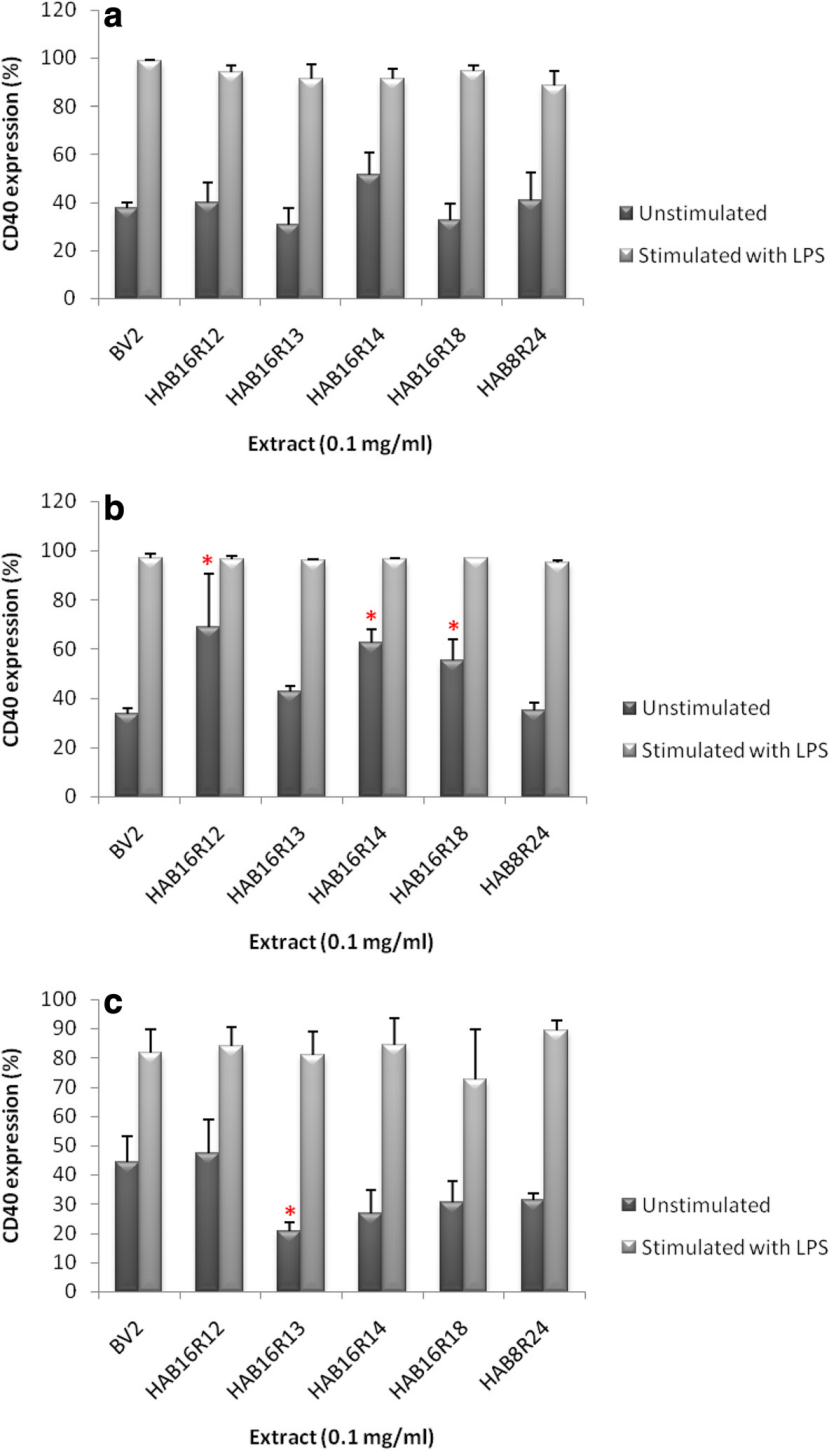
Effect of endophytic extracts on CD40 expression of BV2 microglia at 18 h (**a**), 24 h (**b**) and 48 h (**c**). Results expressed in percentage of BV2 CD40 are mean ± SEM of three independent experiments. *P < 0.05 when compared to control group (BV2)

The subtoxic extracts (0.1 mg/mL) were examined for their suppressive effects against CD40 expression on LPS-stimulated BV2 microglia cells. BV2 cells expressed CD40 at resting state and the expression of this antigen was found to increase following stimulation with LPS (Additional file 1: Figure S2). In general, the extracts did not significantly alter CD40 expression in activated BV2 microglia cells. The extracts-induced alteration of CD40 expression, however, was observed amongst BV2 cells at resting state. At 24 h, extracts HAB16R12, HAB16R14 and HAB16R18 significantly (*P* < 0.05) increased CD40 expression on unstimulated BV2 cells [Fig. 3(b)]. On the other hand, 48 h incubation with extract HAB16R13 significantly (*P* < 0.05) suppressed CD40 expression on unstimulated BV2 cells by 53.4 % (Fig. 3(c)).

### Endophytic extracts suppressed IL-6 and TNF-α in stimulated BV2 microglia cells

In the CNS, proinflammatory cytokines are produced primarily from activated microglia and are involved in the pathogenesis of brain inflammation [1]. In order to investigate the effects of endophytic extracts (0.1 mg/ml) on production of pro- and anti-inflammatory molecules, the amounts of IL-6, IL-10, IL-12p70, TNF-α, MCP-1 and IFN-γ in treated BV2 microglia cells were measured using a FACSCalibur flowcytometer. IL-6 level in unstimulated BV2 microglia cells were below the limit of detection (5 pg/mL). Cells stimulated with LPS, on the contrary, produced high IL-6 (Table 1). All five extracts significantly (*P* < 0.05) suppressed IL-6 production (47.5–87.3 %) with extract HAB16R12 showing the greatest effect (84.2 % and 87.3 % at 24 h and 48 h, respectively) (Table 1; *P* < 0.05). Similar trend was also observed with TNF-α production. All five extracts reduced TNF-α (50.7–89.7 %) with extract HAB16R14 yielding the greatest suppression (89.7 % and 81.3 %) at 24 h and 48 h (Table 2; *P* < 0.05). The extracts, however, elicited no significant effect against IL-10, IL-12p70 and IFN-γ in BV2 microglia cells (data not shown). BV2 microglia cells exhibited very low expression of these cytokines (<45 pg/mL) be it in the presence (stimulated by) or absence (unstimulated by) of LPS. For MCP-1, the levels produced by the microglia cells were beyond the detectable range of this assay.

**Table 1:** Effect of endophytic extracts (0.1 mg/mL) on the secretion of IL-6 in BV2 microglia cells^1^

	IL-6 production (pg/ml)	
	Stimulated with LPS	
	24 h ± SEM	48 h ± SEM
BV2	3335.90 ± 935.3	3295.36 ± 1171.4
HAB16R12	528.02 ± 308.0*	417.13 ± 229.5*
HAB16R13	1750.67 ± 325.1*	1513.50 ± 385.7*
HAB16R14	1151.70 ± 92.3*	1058.67 ± 46.1*
HAB16R18	1463.44 ± 189.5*	1327.50 ± 160.8*
HAB8R24	745.14 ± 344.2*	652.32 ± 298.5*

^1^Data are expressed as means ± SEM of three independent experiments. *P < 0.05, significantly different when compared to LPS-treated control (BV2)

**Table 2:** Effect of endophytic extracts (0.1 mg/mL) on the secretion of TNF-α in BV2 microglia cells^1^

	TNF-α production (pg/mL)			
	Unstimulated		Stimulated with LPS	
	24 h ± SEM	48 h ± SEM	24 h ± SEM	48 h ± SEM
BV2	33.84 ± 3.3	45.78 ± 13.5	4102.62 ± 999.7	2627.97 ± 432.3
HAB16R12	42.44 ± 21.7	61.80 ± 29.8	1150.71 ± 701.4*	1050.06 ± 577.7*
HAB16R13	43.33 ± 1.9	79.82 ± 2.7	960.88 ± 222.7*	982.20 ± 134.8*
HAB16R14	23.83 ± 4.6	58.51 ± 1.2	424.63 ± 98.0*	490.96 ± 86.7*
HAB16R18	32.22 ± 6.1	73.15 ± 0.9	1072.08 ± 332.7*	1052.11 ± 193.7*
HAB8R24	77.28 ± 6.4	103.56 ± 9.1	1511.54 ± 174.5*	1295.21 ± 265.6*

^1^Data are expressed as means ± SEM of three independent experiments. *P < 0.05, significantly different when compared to LPS-treated control (BV2)

## Discussion

Efforts in the past decade have been made to develop anti-inflammatory agents that are capable of inhibiting microglia activation and preventing neuronal cell death. The long-term use of the anti-inflammatory drugs is limited by of their side effects. As such, there have been many attempts uncover alternative anti-inflammatory agents from natural sources. There were already reports of anti-inflammatory activity by metabolites of several fungal and actinomycetous endophytes using *in vitro* and *in vivo* model. Phomol, 4-arylcoumarins and ergoflavin produced by endophytes showed anti-inflammatory activity using reporter gene assays and in ear oedema model in mice [17], murine macrophage RAW 264.7 cells [18] and human monocytic cell line [19], respectively. None of the reports, however, documented the anti-inflammatory activity of endophytes aganinst activated microglia cells. Thus, this is the first attempt to investigate anti-inflammatory properties of endophytes using an *in vitro* model of microglia cells.

*Cytospora sp.* has been previously reported to produce antibiotics: cytosporacin [20], grahamimycin A [21], cytoskyrin A [22], cytosporone E [23] and cytosporic acid [24]. Apart from antibacterial effect, cytosporic acid exhibited antiviral effect. It inhibited a critical enzyme involved in the replication of HIV with an IC_50_ of 20 μM [24]. The pure compound cytoskyrin A displayed poor cytotoxicity against some tumour cell lines (IC_50_ > 5 μg/ml) *in vitro* [23]. Grahamimycin A, also an anti-fungal agent, did not induce any toxic symptoms in adult mice [21]. To date, reports on anti-inflammatory effect of these compounds are very limited. The association of these compounds with the anti-inflammatory effect of the tested Malaysian endophytic fungal extracts (HAB16R12, HAB16R13, HAB16R14, HAB16R18 and HAB8R24) remains unclear.

Inflammation is closely related to pathogenesis of neurodegenerative diseases like Alzheimer’s disease [25], Parkinson’s disease [26] and multiple sclerosis, as well as cerebral ischaemia and post traumatic brain injuries [27]. Microglia cells are mainly responsible for inducing inflammation in the CNS. Uncontrolled activation of microglia which could result in quantitative release of various inflammatory mediators such as oxygen free radicals and nitric oxide (NO) has been shown to be directly toxic to neurons [28]. Intervention against the activation process of microglia may therefore become a promising therapy for the treatment of many neurodegenerative conditions.

In this study, the anti-inflammatory effect of endophytic extracts was investigated against LPS stimulated microglia. These extracts were previously found to exhibit excellent BACE1 inhibitory activity [11] and here their protective effects against inflammation induced NO and pro-inflammatory cytokines were demonstrated. It is known that activated microglia release NO and this process has been implicated as an important mediator in the processes of CNS inflammation [27]. The present study showed that pre-treatment with all five endophytic extracts (0.1 and 1.0 mg/ml dose) significantly (*p* < 0.05) lowered NO production in LPS-activated BV2 cells with no significant effect on basal NO release by the cells. Extracts at 1.0 mg/mL, however, was toxic to BV2 cells and the reduced NO levels for cells pre-treated at this dosage was likely attributed to the pronounced cell loss. Interestingly, pre-treatment with extracts at 0.1 mg/ml was generally more effective than L-NAME, an iNOS inhibitor, in lowering NO production in activated microglia. The mechanism underlying the NO lowering effects by endophytic extracts remains poorly understood. There is, however, a large number of studies have considered that the antagonistic effects of natural compounds on NO production are due to suppression of NFĸB [29].

Microglial cells express the costimulatory molecule CD40 upon activation [30–32]. CD40 is considered as a member of the tumour necrosis factor receptor (TNF-R) superfamily and is expressed as a 45–50 kD cell surface molecule. Interaction between CD40 on microglia cells and its ligand on T lymphocytes triggers a series of intracellular signaling events that further propagate inflammation [33]. The relationship between the pharmacological effects of the present tested endophytic extracts with CD40 expression on BV2, be it at resting or activated state, remains unclear and requires further investigations. This is because the extracts, which were found to affect baseline expression of CD40, exhibited no effect against elevated expression of the said antigen on activated cells. The overall effect of the tested endophytic extracts against CD40 expression on unstimulated BV2, in particular, was bizarre and failed to exhibit an apparent trend. Three endophytic extracts (HAB16R12, HAB16R14 and HAB16R18) increased the expression of the said antigen on cells at resting state. The changes, however, appeared to be temporary (24 h) as the elevated expression of CD40 on treated cells was found to be restored to baseline level following prolonged incubation (48 h). Yet another extract, HAB16R13, significantly attenuated CD40 expression on unstimulated BV2 cells after 48 h. Nevertheless, given none of the extracts significantly affect CD40 expression on activated microglia, the present study postulates that the anti-inflammatory effect as induced by the endophytic extracts could be independent of CD40 expression. Activated microglial cells are also known to highly express other biochemical markers like CD 11c, CD68 and LN-3 [34]. It may be worth exploring the correlation the extracts with these markers in the future.

Besides NO, excessive production of inflammatory mediators such as proinflammatory cytokines including interleukin-1 beta (IL-1β) and tumor necrosis factor (TNF) from activated microglia contributes to uncontrolled inflammation in neurodegenerative diseases [35]. Here, the effects of five endophytic extracts against various inflammatory cytokines (IL-6, IL-10, IL-12p70, TNF-α, and IFN-γ) and chemokine (MCP-1) were studied. At 0.1 mg/mL, all extracts inhibited expression of proinflammatory cytokines, namely IL-6 and TNF-α in LPS-activated microglia. The extracts, however, exhibited no effect against the rest of the proinflammatory cytokines (IL-10, IL-12p70 and IFN-γ). The results from the present study also showed that BV2 cells itself did not up-regulate these cytokines even after LPS stimulation. TNF-α and IL-6 are amongst the main proinflammatory cytokines that are produced by activated microglia during CNS inflammation [36]. Indeed, these cytokines are abundantly induced in brain microglia by stimulants such as LPS [37], IFN-γ, amyloid-β [38]. TNF-α and IL-6 appear to play a dual role in brain injury and neurodegeneration that include both neurotrophic and neurotoxic effects [39].

A potential anti-inflammatory role of these endophytic fungal extracts on microglia is indicated. Although this study was performed on LPS-stimulated BV2 microglia cells, this paradigm partially reflects the pathological condition where activated microglia influences neuron viability [35]. Further studies are required to fully assess the limiting effects of these extracts on microglia functionality and the ability of such metabolites to cross the blood brain barrier and remain effective.

## Conclusions

In conclusion, the results demonstrated that these endophytic extracts exerted inhibitory effect against NO and proinflammatory cytokines (IL-6 and TNF-α) produced by activated BV2 microglia. It is likely that they would have a neuroprotective effect against inflammation resulting from the presence of inflammatory mediators such as NO, IL-6 and TNF-α in activated BV2 microglia. To our knowledge, this is the first report of the effect of a fungal endophytic extract in controlling inflammation in BV2 microglia cells.

## Additional file

Additional file 1: Figure S1. As a reference, cells were pre-treated only with endophytic extracts HAB16R12 (a), HAB16R13 (b), HAB16R14 (c), HAB16R18 (d) and HAB8R24 (e) for 18 h, 24 h and 48 h, and the culture supernatant subjected to nitrite quantification. Data are expressed as means ± SEM of three independent experiments. **Figure S2**: CD40 expression of unstimulated and LPS-stimulated BV2 microglia as determined by immunophenotyping.
