# Supplementary material for: Malaysian endophytic fungal extracts-induced anti-inflammation in Lipopolysaccharide-activated BV-2 microglia is associated with attenuation of NO production and, IL-6 and TNF-α expression
Source: BMC Complement Altern Med. 2015 Jun 6;15:166. doi: 10.1186/s12906-015-0685-5 (PMC4457982; doi:10.1186/s12906-015-0685-5)
Supplement: Additional file 1: Figure S1. — As a reference, cells were pre-treated only with endophytic extracts HAB16R12 (a), HAB16R13 (b), HAB16R14 (c), HAB16R18 (d) and HAB8R24 (e) for 18 h, 24 h and 48 h, and the culture supernatant subjected to nitrite quantification. Data are expressed as means ± SEM of three independent experiments. Figure S2: CD40 expression of unstimulated and LPS-stimulated BV2 microglia as determined by immunophenotyping. [file 12906_2015_685_MOESM1_ESM.doc]

a


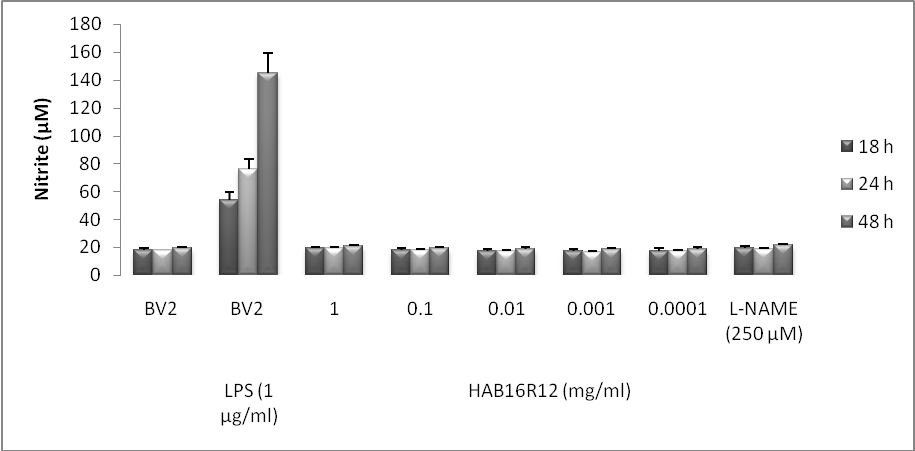


b


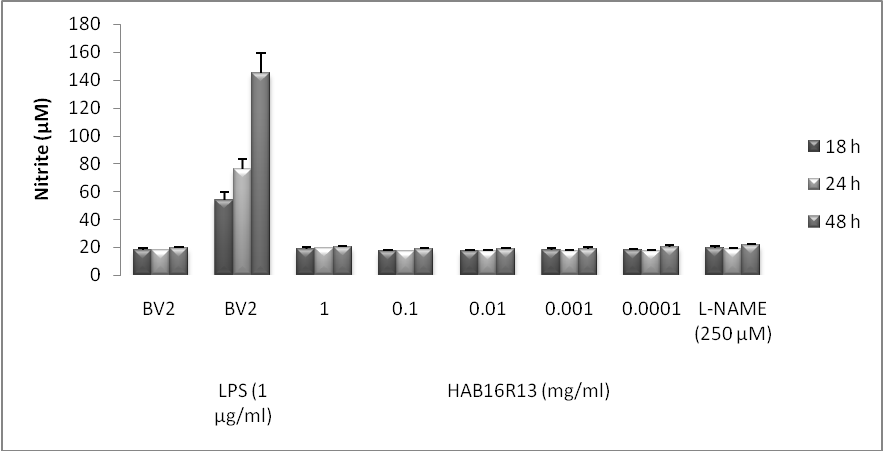


c


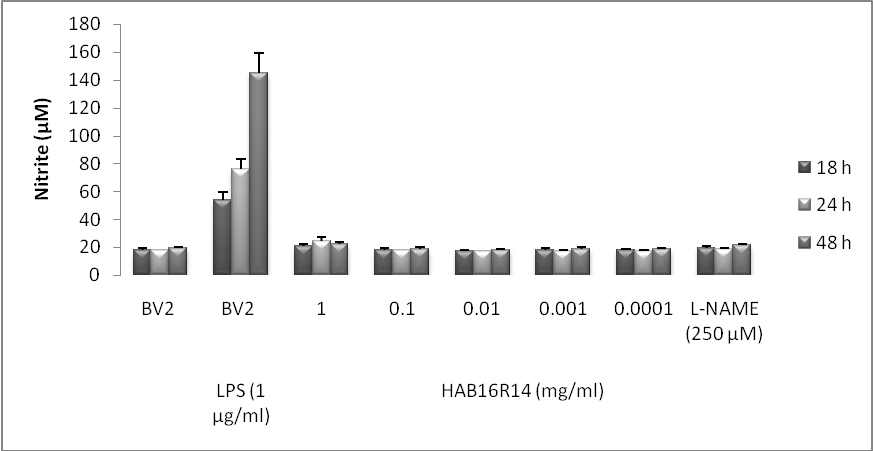


d


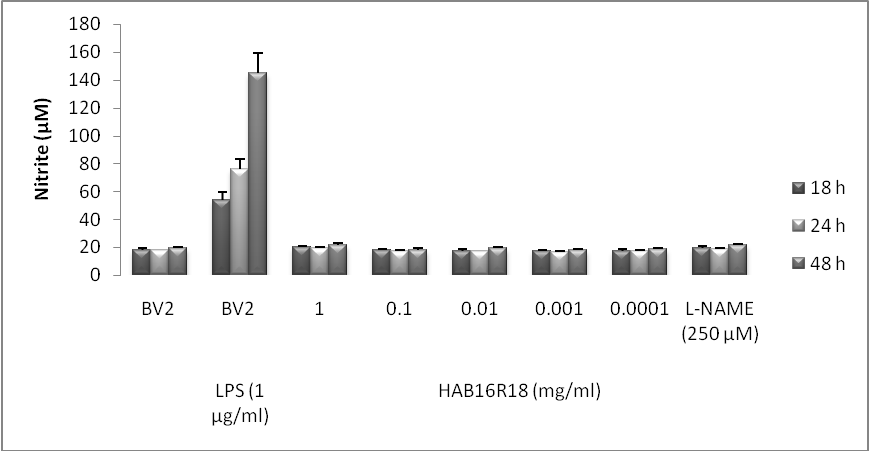


e


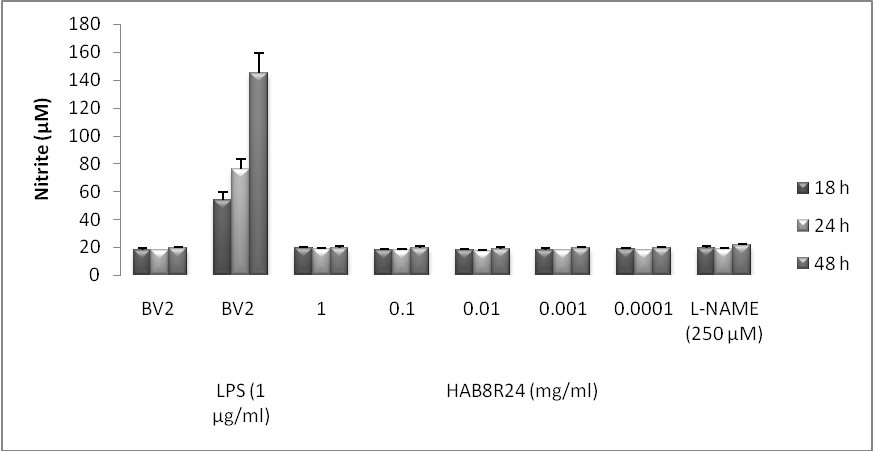


FIGURE S1: As a reference, cells were pre-treated only with endophytic extracts HAB16R12 (a), HAB16R13 (b), HAB16R14 (c), HAB16R18 (d) and HAB8R24 (e) for 18 h, 24 h and 48 h, and the culture supernatant subjected to nitrite quantification. Data are expressed as means ± SEM of three independent experiments.

| Unstimulated | Stimulated with LPS |
| --- | --- |
| 42.74% | 99.24% |

FIGURE S2: CD40 expression of unstimulated and LPS-stimulated BV2 microglia as determined by immunophenotyping.
